# Correlation Between Immune-Related Adverse Events and Prognosis in Hepatocellular Carcinoma Patients Treated With Immune Checkpoint Inhibitors

**DOI:** 10.3389/fimmu.2021.794099

**Published:** 2021-12-07

**Authors:** Shuo Xu, Ruixue Lai, Qian Zhao, Pandong Zhao, Ruili Zhao, Zhanjun Guo

**Affiliations:** ^1^ Department of Rheumatology and Immunology, The Fourth Hospital of Hebei Medical University, Shijiazhuang, China; ^2^ Department of Otolaryngology, The Fourth Hospital of Hebei Medical University, Shijiazhuang, China

**Keywords:** hepatocellular carcinoma, immune-related adverse events, immune checkpoint inhibitors, anti-PD-1 antibody, OS, PFS

## Abstract

**Background:**

Immune-related adverse events (irAEs) caused by immune checkpoint inhibitors (ICIs) were associated with clinical benefit in cancer patients of melanoma, a lung cancer. In the present study, we investigated the correlation between irAE and ICI efficacy in hepatocellular carcinoma (HCC) patients.

**Methods:**

We divided the HCC patients who received the anti-PD-1 antibody into two groups as irAE group and non-irAE group according to the National Cancer Institute Common Terminology Criteria for Adverse Events ver. 4.03. The treatment efficacy of ICIs was evaluated with objective response rate (ORR), disease control rate (DCR), progression-free survival (PFS), and overall survival (OS).

**Result:**

Of the 65 HCC patients who received the anti-PD-1 antibody (monotherapy or combined with targeted medicine), median PFS in the irAE group was superior to that in the non-irAE group (302 days vs. 148 days, *p* = 0.004). Median OS in the irAE group was also better than that in the non-irAE group (374 days vs. 279 days, *p* = 0.038). Although the statistical difference for DCR in the irAE group and non-irAE group was not reached, the DCR of the irAE displayed a trend better than that of the non-irAE group (41.20% vs. 20.80%, *p* = 0.118). Multivariate analysis also demonstrated that the non-irAE group (HR = 6.410, 95% CI: 1.404 to 29.275) was associated independently with the poor prognosis.

**Conclusions:**

Development of irAEs was associated with clinical benefit for HCC patients who were treated with immune checkpoint inhibitors; irAE, particularly low-grade irAE, was a predictable marker for better ICI treatment efficiency in HCC patients.

## Introduction

Hepatocellular carcinoma (HCC) is an important global health issue (1), with approximately 906,000 new cases and 830,000 deaths, making it the sixth most commonly cancer and the third leading cause of cancer death (2). The first-line treatment for unresectable HCC included multi-targeted tyrosine kinase inhibitors (TKI) sorafenib and lenvatinib (3–5), the second-line therapies including regorafenib, cabozantinib, and ramucirumab (6–8), but these drugs prolonged the HCC lifespan with a limited increase.

Immune checkpoint inhibitors (ICIs) have recently been a source of promising new cancer treatments that increase the cytotoxicity of T cells by blocking intrinsic downregulators of immunity such as cytotoxic T-lymphocyte antigen 4 (CTLA-4) and programmed cell death 1 (PD-1) or its ligand of programmed cell death ligand 1 (PD-L1) so as to enhance antitumor activity (9). Recently, ICIs have made a breakthrough in a range of tumor therapies such as advanced non-small-cell lung cancer (NSCLC) (10), melanoma (11), renal cell carcinoma (12), and squamous cell carcinoma of head and neck (SCCHN) (13). In the early phase I/II or phase II studies, the objective response rate (ORR) for advanced HCC people who were treated with the anti-PD-1 antibody of nivolumab or pembrolizumab has been shown to be 14%–20% with prolonged PFS and OS (14–16). However, in the further phase III trials, no satisfying results were obtained for these two antibodies (17, 18). The IMbrave150 trial combining the anti-PD-L1 antibody of atezolizumab and the anti-VEGF antibody of bevacizumab obtained a breakthrough for advanced HCC treatment referring to ORR, PFS, and OS when compared with sorafenib as the first-line treatment (19–21). In addition, the combination of lenvatinib plus pembrolizumab as a front-line treatment in advanced HCC patients is being evaluated based on its good treatment efficiency in a Phase I study (22, 23). In spite of the breakthrough for ICIs on advanced HCC treatment, only a few biomedical predictors of response to ICIs such as microsatellite instability (MSI) and gut microbiome were identified (24, 25).

Immune checkpoint inhibitors cause imbalances in immunological tolerance, resulting in inflammatory side effects which are called immune-related adverse events (irAEs) (26). It needs frequent monitoring and immunosuppressive and endocrine therapy based on the severity of the respective AE (11, 27–29). The immune-related adverse events are believed to be related to the role that immune checkpoints play in maintaining immunologic homeostasis. The occurrence of irAEs may be related to the following potential mechanisms: increasing T-cell activity against antigens that are present in tumors and healthy tissue, increasing levels of preexisting autoantibodies, an increase in the level of inflammatory cytokines (such as interleukin-17), and enhanced complement-mediated inflammation due to direct binding of an antibody against cytotoxic T-lymphocyte antigen 4 (CTLA-4) (9). However, the precise pathophysiology underlying irAEs still needs to be further explored. IrAEs affect almost every organ of the body and are most commonly observed in the skin, gastrointestinal tract, lung, and endocrine, musculoskeletal, and other organs (30).

Recently, several studies have shown that irAEs were associated with efficacy of anti-PD-1 antibody treatment in patients with melanoma, non–small cell lung cancer, and gastric cancer (31–33). In contrast, few data are available considering the relationship between irAE and treatment efficacy of immunotherapy for HCC. In the present study, we investigated the correlation between irAEs and ICIs efficacy in HCC patients.

## Methods

### Patients

HCC patients with disease stage III or IV who were treated with the anti-PD-1 antibody (monotherapy or combined with targeted medicine) between January 2019 and April 2020 at the Fourth Hospital of Hebei Medical University were involved for analysis. Patients who received other previous treatments with immunotherapy were excluded. We reviewed the medical records, and the following characteristics of patients were collected: age, gender, Eastern Cooperative Oncology Group performance status (ECOG PS), treatment line number, TNM, alpha fetoprotein (AFP), portal vein tumor thrombus (PVTT), number of tumors, history of transarterial chemoembolization (TACE) or surgery, concurrent therapy, metastatic status, history of hepatitis B virus (HBV), baseline blood cell count, prothrombin time (PT), max tumor size, alanine aminotransferase (ALT), aspartate aminotransferase (AST), and albumin and total bilirubin before initiating treatment. The neutrophil-to-lymphocyte ratio (NLR) was calculated by dividing the lymphocyte count into neutrophil count. IrAEs were defined as those inflammatory side effects which were caused by imbalances in immunological tolerance because of the immune checkpoint inhibitors. For the irAE assessment, the National Cancer Institute Common Terminology Criteria for Adverse Events ver. 4.03 was used (https://ctep.cancer.gov/protocolDevelopment/electronic_applications/ctc.htm#ctc_40). We divided the patients into two groups, irAE group and non-irAE group, based on occurrence of irAEs.

The study protocol was reviewed and approved by the ethics committee of the Fourth Hospital of Hebei Medical University. Due to the retrospective nature of this study, informed consent was not obtained from each patient.

### Treatment and Assessment

Patients received a standard anti-PD-1 antibody (monotherapy or combine with targeted medicine) every 3 weeks until disease progression, clinical deterioration, unacceptable toxicity, or patient’s refusal. Types of immunotherapy drugs received were camrelizumab, sintilimab, pembrolizumab, and toripalimab, whereas the targeted medicines were apatinib, sorafenib, and lenvatinib (**Table S1**). Objective tumor response was evaluated by computed tomography (CT) or magnetic resonance imaging (MRI) scan repeated every 2 or 3 cycles according to the RECIST version 1.1.

### Statistical Analysis

Differences between the two groups were compared using the Fisher’s exact tests for classified variables and Mann–Whitney U tests for continuous variables. The result of continuous variables was held up as median (interquartile range (IQR)). PFS was defined as the time from the beginning of treatment to progression or death from any cause; in addition, the PFS of patients who survived without disease progression at the end of follow-up was defined as the time from the beginning to the ending date of follow-up. OS was measured until death or censored at the latest follow-up for surviving patients. Probabilities of survival were estimated using the Kaplan–Meier method and compared using the log-rank test. Univariate analysis and multivariate analysis using a Cox proportional hazard regression model were performed to explore prognostic factors for survival. All statistical analyses were performed using SPSS (IBM SPSS 20.0, NY, USA). All p values are two-sided, and *p* < 0.05 was considered to indicate a statistically significant difference.

## Results

### Patient Characteristics

Sixty-nine HCC patients treated with the anti-PD-1 antibody (monotherapy or combine with targeted medicine) were identified as the research subjects. Among them, five patients were excluded because of loss to follow-up and 37 (56.9%) patients died during the follow-up. The clinical characteristics are listed in **Table 1**. The median OS and median PFS were 279 days (95% CI, 268 to 291 days) and 180 days (95% CI, 140 to 220 days) for overall patients. As shown in **Table 2**, partial response (PR) was achieved in four patients and stable disease (SD) was observed in 13 patients, which resulted in an ORR of 6.2% (95% CI: 0.2% to 12.2%) and DCR of 26.2% (95% CI: 15.2% to 37.1%).

**Table 1:** Characteristics of patients in irAE and non-irAE groups.

	Total no. (%)	Non-irAE no. (%)	irAE no. (%)	*p*-value
Total N	65	48	17	
Gender				
Female	20 (30.8)	10 (20.8)	10 (58.8)	0.006
Male	45 (69.2)	38 (79.2)	7 (41.2)	
Age				
<65	51 (78.5)	40 (83.3)	11 (64.7)	0.167
≥65	14 (21.5)	8 (16.7)	6 (35.3)	
ECOG PS				
≤1	44 (67.7)	33 (68.8)	11 (64.7)	0.770
>1	21 (32.3)	15 (31.2)	6 (35.3)	
Treatment line				
≤2	59 (90.8)	42 (87.5)	17 (100.0)	0.327
≥3	6 (9.2)	6 (12.5)	0 (0.0)	
TNM				
III	33 (50.8)	23 (47.9)	10 (58.8)	0.574
IV	32 (49.2)	25 (52.1)	7 (41.2)	
AFP				
<400	41 (63.1)	30 (62.5)	11 (64.7)	1.000
≥400	24 (36.9)	18 (37.5)	6 (35.3)	
PVTT				
No	18 (27.7)	14 (29.2)	4 (23.5)	0.760
Yes	47 (72.3)	34 (70.8)	13 (76.5)	
Tumor number				
<3	41 (63.1)	24 (50.0)	17 (100.0)	0.001
≥3	24 (36.9)	24 (50.0)	0 (0.0)	
Concurrent therapy				
No	11 (16.9)	8 (16.7)	3 (17.6)	1.000
Combine targeted drugs	54 (83.1)	40 (83.3)	14 (82.4)	
Prior therapy				
No	25 (38.5)	24 (50.0)	1 (5.9)	0.001
TACE/surgery	40 (61.5)	24 (50.0)	16 (94.1)	
Metastasis				
No	27 (41.5)	20 (41.7)	7 (41.2)	1.000
Yes	38 (58.5)	28 (58.3)	10 (58.8)	
HBV infection				
No	8 (12.3)	6 (12.5)	2 (11.8)	1.000
Yes	57 (87.7)	42 (87.5)	15 (88.2)	
Child–Pugh score				
A	33 (50.8)	21 (43.8)	12 (70.6)	0.214
B	24 (36.9)	20 (41.7)	4 (23.5)	
C	8 (12.3)	7 (14.6)	1 (5.9)	
NLR				
Low (≤4)	26 (40.0)	19 (39.6)	7 (41.2)	1.000
High (>4)	39 (60.0)	29 (60.4)	10 (58.8)	
Clinical baseline value (median (IQR))				
Plt	149.00 (123.00, 166.00)	149.00 (134.00, 158.00)	98.00 (67.00, 180.00)	0.081
WBC	3.80 (3.33, 5.28)	3.80 (3.34, 5.04)	3.33 (3.05, 5.89)	0.293
Neutrophil	2.92 (2.43, 3.81)	2.92 (2.43, 3.68)	2.44 (2.04, 4.48)	0.653
Lymphocyte	0.61 (0.52, 0.65)	0.60 (0.52, 0.74)	0.61 (0.46, 0.65)	0.207
NLR	4.58 (3.48, 5.62)	4.58 (3.20, 5.62)	4.16 (3.75, 9.88)	0.869
Hemoglobin	129.00 (111.80,137.00)	132.40 (111.80,137.00)	121.5 (108.0,139.50)	0.167
Max. tumor size	6.00 (4.00, 9.50)	7.00 (5.00, 9.00)	4.00 (3.00, 10.00)	0.090
PT	12.70 (11.90, 13.35)	12.60 (11.90, 12.80)	12.90 (11.85, 14.45)	0.558
AST	65.90 (43.50, 72.20)	65.90 (48.50, 72.20)	42.20 (30.80, 59.10)	0.030
ALT	31.10 (23.50, 59.20)	36.95 (23.15, 87.80)	31.10 (23.30, 48.70)	0.099
Albumin	37.70 (32.95, 42.05)	37.10 (30.45, 42.05)	39.60 (34.30, 44.80)	0.307
Total bilirubin	18.90 (15.70, 44.25)	31.70 (15.85, 49.40)	17.00 (15.70, 42.60)	0.145

ECOG PS, Eastern Cooperative Oncology Group performance status; AFP, alpha fetoprotein; PVTT, portal vein tumor thrombus; TACE, transarterial chemoembolization; HBV, hepatitis B virus; PT, prothrombin time; ALT, alanine aminotransferase; AST, aspartate aminotransferase; NLR, neutrophil-to-lymphocyte ratio.

**Table 2:** Response to immunotherapy.

Response	Total	irAE group	Non-irAE group	*p*-value
PD	48	10	38	-
SD	13	3	10	-
PR	4	4	0	-
CR	0	0	0	-
ORR	6.2% (95% CI: 0.2% to 12.2%)	23.5% (95% CI: 1% to 46%)	-	-
DCR	26.2% (95% CI: 15.2% to 37.1%)	41.2% (95% CI: 15.1% to 67.3%)	20.80% (95% CI: 8.9% to 32.8%)	0.118

SD, stable disease; PR, partial response; PD, progressive disease; CR, complete response; ORR, objective response rate; DCR, disease control rate.

### Comparison Between irAE and Non-irAE Groups

The patient backgrounds of the irAE and non-irAE groups are summarized in **Table 1**. No significant differences in clinical profiles, apart from gender, number of tumors, history of previous treatment, and AST were observed between the two groups.

An ORR of 23.5% (95% CI: 1% to 46%) and a DCR of 41.20% (95% CI: 15.1% to 67.3%) were observed for all 17 irAE patients (4 PR, 3 SD), whereas only 10 SD patients with DCR of 20.80% (95% CI: 8.9% to 32.8%) were achieved in the non-irAE group. Although DCR in the irAE group was superior to that in the non-irAE group, it was not statistically significant (*p* = 0.118 > 0.05).

**Figure 1:**
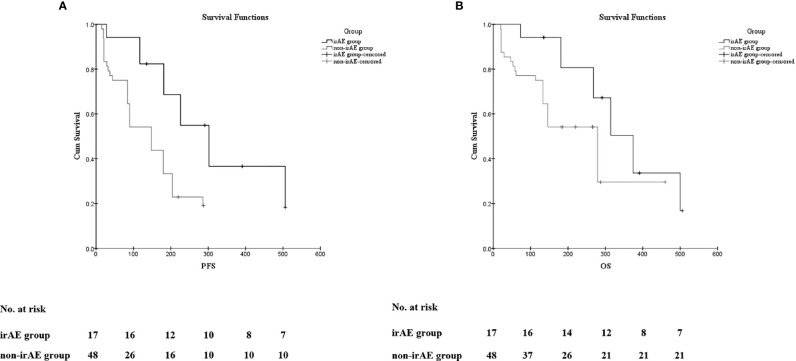
The association of irAEs on the prognosis of HCC patients. **(A)** The Kaplan–Meier curve of progression-free survival (PFS). **(B)** The Kaplan–Meier curve of overall survival (OS).

The Kaplan–Meier curves of PFS and OS in the irAE and the non-irAE groups are shown in **Figure 1**. The median PFS of 302 days (95% CI: 201 to 402 days) for the irAE group was significantly extended compared with the median PFS of 148 days (95% CI: 104 to 191 days) for the non-irAE group (*p* = 0.004) (**Figure 1A**). In addition, the median OS was 374 days (95% CI: 291 to 456 days) in the irAE group and 279 days (95% CI: 205 to 352 days) in the non-irAE group (*p* = 0.038) (**Figure 1B**). All of these indicated that irAEs associated with prolonged median PFS and median OS for HCC patients with ICIs treatment.

In addition, we divided the patients with irAEs into two groups: irAE-A group (patients with endocrine, dermatological, or gastrointestinal irAEs) and irAE-B group (patients with other irAEs). The median PFS and median OS of the two groups mentioned above and the non-irAE group were compared pairwise. As shown in **Table 3**, it is obvious that the median PFS and median OS of the people who experienced endocrine, dermatological, or gastrointestinal irAEs had obvious advantages compared with those who experienced no irAEs (median PFS: 302 vs. 148 days, *p* = 0.002; median OS: 374 vs. 279 days, *p* = 0.034).

**Table 3:** Kaplan–Meier survival curve of progression-free survival (PFS) and overall survival (OS).

	Median PFS (days)	*p-*value	Median OS (days)	*p*-value
irAE group vs. non-irAE group				
irAE group	302 (95% CI: 201 to 402)	0.004	374 (95% CI: 291 to 456)	0.038
Non-irAE group	148 (95% CI: 104 to 191)		279 (95% CI: 205 to 352)	
irAE-A group vs. non-irAE group				
irAE-A group	302 (95% CI: 206 to 398)	0.002	374 (95% CI: 295 to 453)	0.034
Non-irAE group	148 (95% CI: 104 to 191)		279 (95% CI: 205 to 352)	
irAE-B group vs. non-irAE group				
irAE-B group	181 (95% CI: 81 to 281)	0.642	314 (95% CI: 114 to 514)	0.613
Non-irAE group	148 (95% CI: 104 to 191)		279 (95% CI: 205 to 352)	
irAE-A group vs. irAE-B group				
irAE-A group	302 (95% CI: 206 to 398)	0.126	374 (95% CI: 295 to 453)	0.198
irAE-B group	181 (95% CI: 81 to 281)		314 (95% CI: 114 to 514)	

PFS, progression-free survival; OS, overall survival; irAE-A group, the group with endocrine, dermatological, or gastrointestinal irAEs; irAE-B group, the group with other irAEs.

In the univariate analysis for OS using gender (male or female), ECOG score, treatment line number (≤2 or ≥3), AFP level (<400 or ≥400), number of tumors (<3 or ≥3), TNM (III or IV), metastatic status (Yes or No), and irAEs status (non-irAE group or irAE group) as covariates in ICI-treated HCC, clinical characteristics including PS > 1, ≥3 treatment line, TNM (IV), AFP ≥ 400, and tumor metastasis were significantly associated with shorter OS at statistical levels. The non-irAE group was associated with shorter OS at borderline statistical levels (HR = 2.295, 95% CI: 0.996 to 5.288, *p* = 0.051). Moreover, multivariate analysis demonstrated that non-irAE (HR = 6.410, 95% CI: 1.404 to 29.275, *p* = 0.017) was independently associated with a poor prognosis (**Table 4**).

**Table 4:** Univariate and multivariate analyses of OS with Cox regression models.

Covariate	Univariate analysis (n = 65)				Multivariate analysis (n = 65)			
	HR	95.0% CI		*p*-value	HR	95.0% CI		*p*-value
Group								
irAE	Reference				Reference			
Non-irAE	2.295	0.996	5.288	0.051	6.410	1.404	29.275	0.017
Gender								
Female	Reference				Reference			
Male	1.044	0.510	2.137	0.906	2.004	0.449	8.934	0.362
ECOG PS								
≤1	Reference				Reference			
>1	4.366	1.533	12.433	0.006	6.896	1.530	31.077	0.012
Treatment line								
≤2	Reference				Reference			
≥3	4.526	1.698	12.066	0.003	2.638	0.453	15.886	0.277
AFP								
<400	Reference				Reference			
≥400	2.765	1.206	6.342	0.016	1.540	0.476	4.987	0.471
Tumor number								
<3	Reference				Reference			
≥3	1.045	0.520	2.100	0.902	1.651	0.600	4.544	0.332
TNM								
III	Reference				Reference			
IV	5.996	2.722	13.211	0.001	5.427	2.323	12.679	0.001
Metastasis								
No	Reference				Reference			
Yes	2.308	1.159	4.597	0.017	3.265	1.141	9.348	0.027

ECOG PS, Eastern Cooperative Oncology Group performance status; AFP, alpha fetoprotein.

### Toxicity

Seventeen of the 65 patients (26.15%) experienced irAEs in our study. Details of these irAEs are shown in **Tables S2** and **S3**. The most frequent adverse event was diarrhea/colitis (n = 6), and the second is hypothyroidism (n = 4). The median time to onset of irAEs was 68 days (range 26–142 days). There was no grade 3 adverse event observed in our research; no people discontinued the anti-PD-1 antibody because of the irAEs.

## Discussion

Our result indicated that irAEs were associated with better treatment efficacy of ICIs in HCC patients referring to median PFS and median OS, which was compatible with treatment efficacy for irAEs in other tumors, such as melanoma, head and neck squamous cell carcinoma, non-small cell lung cancer, renal cell carcinoma, and urothelial carcinoma (32, 34). We could analyze only a limited number of patient samples, but it is speculated that occurrence of irAEs may be associated with survival even after adjusting other prognostic factors in HCC patients treated with the anti-PD-1 antibody. Although DCR or ORR had the advantage in the irAE group, it did not show a statistical difference due to the small sample size. In the previous meta-analysis (35), the occurrences of endocrine, dermatological, and gastrointestinal irAEs were significantly associated with a favorable prognosis in patients treated with ICIs, but other irAEs were not. The occurrence of low-grade, not the high-grade, irAEs was significantly associated with a favorable OS in patients receiving ICIs. Therefore, it is considered reasonable to divide the irAE group into irAE-A group (patients with endocrine, dermatological, or gastrointestinal irAEs) and irAE-B group (patients with other irAEs) and compare them with the non-irAE group in pairs for survival analysis. Our analysis showed that the patients with diarrhea/colitis, hyperthyroidism/hypothyroidism, or rash had a better prognosis. The combined immunotherapy would be mainstream for advanced cancer treatment in the future, while bringing considerable benefits; it will also bring more side effects. Comprehensive judgment based on big data analysis combined with patients’ genetic background and different irAE types would be the key to individualized precision therapy.

In our research, there were no grade 3 to 5 adverse events related to the anti-PD-1 antibody and no exacerbation of irAEs after detection, which is probably due to our baseline examination including liver and kidney function, pituitary function, thyroid function, cardiac function, and electrocardiogram (ECG), as well as chest CT every 3 weeks so as to treat irAEs at the early stage. Our results implied that irAEs particularly low-grade irAEs were a predictable marker for better ICI treatment efficiency in HCC patients; we might recommend continuing use for ICI therapy in low-grade irAEs patients. We would not be worried about the occurrence of irAEs in the clinical treatment but exert our utmost effort to prevent fatal irAEs such as immune-associated myocarditis and adrenocortical dysfunction. We would not resist the low-grade irAEs because they provide clinical benefit with low clinical risk.

The occurrence of irAEs during immunotherapy may suggest a higher response rate, but the efficacy of ICIs remains to be limited with an ORR rate no more than 40% even for combined ICI therapy (36). Consequently, it is critical to identify the dominant population and prognostic indicators of ICIs. Some recommended predictive indexes for efficacy include PD-L1 expression, tumor mutation burden (TMB), microsatellite instability-high (MSI-H), and gut microbiome (25, 37, 38). However, the biomarkers for irAEs are still blank, and the occurrence of irAEs is usually detected only by regular detection of relevant indicators and clinical observation.

## Limitations

This study has some limitations. First, the study is retrospective and conducted only in one center; the data from a multicenter should be evaluated in the further study. Second, the sample size was small; it may lead to large confidence intervals and affect the imprecision of the results. The larger sample size was valuable for stratified analysis to identify different types of irAEs responsible for ICI efficiency. Third, the effect of combined medicine on adverse reactions and prognosis in patients receiving combined medicine was not excluded in this study, although the combined medicine displayed a spectrum of different adverse events compared with ICIs for HCC treatment. The most common side effects for the systemic treatment of sorafenib and lenvatinib were hypertension, fatigue, proteinuria, and nausea, whereas the most common irAE involved the skin, followed by the gastrointestinal tract and endocrine system (39). Fourth, our study only included HCC patients with no preexisting autoimmune disease; the correlation between irAEs and prognosis in patients with preexisting autoimmune disease needs further exploration. Most immune-related side effects are managed without permanent drug discontinuation for ICI-treated patients complicated with preexisting autoimmune disease, but patients with myasthenia gravis, multiple sclerosis, and inflammatory bowel disease (IBD) might have a higher risk of relapse and exacerbation (16, 40). However, to the best of our knowledge, this is the first work to reveal an association between irAEs and the efficacy of ICIs in HCC. Our goal is to identify potential predictive value for irAEs on treatment efficiency of ICIs from this pilot project first and to build the foundation for future research with a larger population. Findings from this study may also inspire other scientists to look for predictive values of irAEs in other tumors with ICI treatment.

## Conclusions

Occurrence of irAEs was significantly associated with better clinical prognosis of HCC patients treated with immune checkpoint inhibitors; irAEs, particularly low-grade irAEs, were a predictable maker for better ICI treatment efficiency in HCC patients.

## Data Availability Statement

The original contributions presented in the study are included in the article/**Supplementary Material**. Further inquiries can be directed to the corresponding authors.

## Ethics Statement

Written informed consent was obtained from the individual(s) for the publication of any potentially identifiable images or data included in this article.

## Author Contributions

All authors listed have made a substantial, direct, and intellectual contribution to the work and approved it for publication.

## Funding

This work was supported by the Natural Science Foundation of China of Hebei Province (Grant No. H2019206428) and the Foundation of Hebei Provincial Department of Science and Technology & Hebei Medical University (Grant No. 2020TXZH03).

## Conflict of Interest

The authors declare that the research was conducted in the absence of any commercial or financial relationships that could be construed as a potential conflict of interest.

## Publisher’s Note

All claims expressed in this article are solely those of the authors and do not necessarily represent those of their affiliated organizations, or those of the publisher, the editors and the reviewers. Any product that may be evaluated in this article, or claim that may be made by its manufacturer, is not guaranteed or endorsed by the publisher.

## Abbreviations

ICIs, immune checkpoint inhibitors; irAE, immune-related adverse event; SD, stable disease; PR, partial response; PD, progressive disease; CR, complete response; ORR, objective response rate; DCR, disease control rate; PFS, progression-free survival; OS, overall survival; HCC, hepatocellular carcinoma; TKI, tyrosine kinase inhibitor; PD-1, programmed cell death 1; CTLA-4, cytotoxic T-lymphocyte antigen 4; NSCLC, non-small-cell lung cancer; SCCHN, squamous cell carcinoma of head and neck; ECOG PS, Eastern Cooperative Oncology Group performance status; AFP, alpha fetoprotein; PVTT, portal vein tumor thrombus; TACE, transarterial chemoembolization; HBV, hepatitis B virus; PT, prothrombin time; ALT, alanine aminotransferase; AST, aspartate aminotransferase; NLR, neutrophil-to-lymphocyte ratio; CT, computed tomography; MRI, magnetic resonance imaging; IQR, interquartile range; ECG, electrocardiogram; TMB, tumor mutation burden; MSI-H, microsatellite instability-high.
