# Supplementary material for: Correlation Between Immune-Related Adverse Events and Prognosis in Hepatocellular Carcinoma Patients Treated With Immune Checkpoint Inhibitors
Source: Front Immunol. 2021 Dec 7;12:794099. doi: 10.3389/fimmu.2021.794099 (PMC8691363; doi:10.3389/fimmu.2021.794099)
Supplement: Supplementary file 1 [file Table_1.docx]

**Table S1** Treatment information of overall patients

| Treatment line | Therapeutic schedule | The number of people |
| --- | --- | --- |
| 1 | Camrelizumab combine Apatinib | 12 |
| 1 | Camrelizumab combine Sorafenib | 6 |
| 1 | Camrelizumab combine Lenvatinib | 3 |
| 1 | Sintilimab combine Lenvatinib | 7 |
| 1 | Pembrolizumab combine Sorafenib | 4 |
| 1 | Pembrolizumab combine Apatinib | 7 |
| 1 | Toripalimab combine Sorafenib | 4 |
| 1 | Pembrolizumab | 2 |
| 1 | Camrelizumab | 1 |
| 2 | Camrelizumab combine Sorafenib | 5 |
| 2 | Camrelizumab combine Apatinib | 1 |
| 2 | Pembrolizumab combine Apatinib | 4 |
| 2 | Toripalimab combine Sorafenib | 1 |
| 2 | Pembrolizumab | 2 |
| 3 | Pembrolizumab | 3 |
| 3 | Camrelizumab | 2 |
| 4 | Camrelizumab | 1 |
