# Supplementary material for: Correlation Between Immune-Related Adverse Events and Prognosis in Hepatocellular Carcinoma Patients Treated With Immune Checkpoint Inhibitors
Source: Front Immunol. 2021 Dec 7;12:794099. doi: 10.3389/fimmu.2021.794099 (PMC8691363; doi:10.3389/fimmu.2021.794099)
Supplement: Supplementary file 2 [file Table_2.docx]

**Table S2** Categorization of irAEs

| irAEs | No. (%) | Median days to onset | Grade of irAEs, n, 1/2/3/4 |
| --- | --- | --- | --- |
| Diarrhea/colitis | 6 (35.29%) | 73.5 | 5/1/0/0 |
| Hypothyroidism | 4 (23.53%) | 86.5 | 3/1/0/0 |
| Creatinine increased | 2 (11.76%) | 42 | 2/0/0/0 |
| Hyperthyroidism | 2 (11.76%) | 36.5 | 2/0/0/0 |
| Rash | 2 (11.76%) | 63.5 | 2/0/0/0 |
| Myalgia | 1 (5.88%) | 29 | 1/0/0/0 |
| AST/ALT/Bilirubin increased | 1 (5.88%) | 53 | 0/1/0/0 |
| Cutaneous hemangioma | 1 (5.88%) | 84 | 1/0/0/0 |
| Myocardial enzyme increased | 1 (5.88%) | 101 | 1/0/0/0 |
