# Supplementary material for: Correlation Between Immune-Related Adverse Events and Prognosis in Hepatocellular Carcinoma Patients Treated With Immune Checkpoint Inhibitors
Source: Front Immunol. 2021 Dec 7;12:794099. doi: 10.3389/fimmu.2021.794099 (PMC8691363; doi:10.3389/fimmu.2021.794099)
Supplement: Supplementary file 3 [file Table_3.docx]

**Table S3** Clinical information of 17 patients in irAE group

|  | irAEs | Grades | Time (days) | Treatment line | Therapeutic schedule | Treatment | Discontinued |
| --- | --- | --- | --- | --- | --- | --- | --- |
| 1 | Creatinine increased | 1 | 58 | 1 | Camrelizumab combine Apatinib | improved renal function | no |
| 2 | Diarrhea/colitis | 1 | 67 | 1 | Sintilimab combine Lenvatinib | observed | no |
| 3 | Hypothyroidism | 1 | 80 | 1 | Camrelizumab combine Sorafenib | observed | no |
| 4 | Cutaneous hemangioma | 1 | 84 | 1 | Camrelizumab combine Apatinib | observed | no |
| 5 | Diarrhea/colitis | 2 | 67 | 1 | Pembrolizumab | observed | no |
| 6 | Hyperthyroidism | 1 | 31 | 1 | Camrelizumab combine Apatinib | observed | no |
|  | Hypothyroidism | 2 | 98 |  |  |  | no |
| 7 | Creatinine increased | 1 | 26 | 1 | Camrelizumab combine Apatinib | improved renal function | no |
| 8 | Diarrhea/colitis | 1 | 142 | 2 | Camrelizumab combine Sorafenib | observed | no |
|  | Myalgia | 1 | 29 |  |  |  | no |
| 9 | Diarrhea/colitis | 1 | 65 | 1 | Sintilimab combine Lenvatinib | observed | no |
| 10 | Rash | 1 | 58 | 1 | Pembrolizumab | observed | no |
| 11 | Diarrhea/colitis | 1 | 95 | 1 | Camrelizumab combine Lenvatinib | observed | no |
| 12 | Hypothyroidism | 1 | 74 | 1 | Pembrolizumab combine Apatinib | observed | no |
| 13 | Rash | 1 | 69 | 1 | Camrelizumab | observed | no |
| 14 | Myocardial enzyme increased | 1 | 101 | 1 | Toripalimab combine Sorafenib | observed | no |
| 15 | Hyperthyroidism | 1 | 42 | 1 | Pembrolizumab combine Sorafenib | observed | no |
|  | Hypothyroidism | 1 | 93 |  |  |  | no |
| 16 | AST/ALT/Bilirubin increased | 2 | 53 | 2 | Toripalimab combine Sorafenib | protect liver treatment | no |
| 17 | Diarrhea/colitis | 1 | 80 | 1 | Sintilimab combine Lenvatinib | observed | no |
